# CCNB1 affects cavernous sinus invasion in pituitary adenomas through the epithelial–mesenchymal transition

**DOI:** 10.1186/s12967-019-2088-8

**Published:** 2019-10-04

**Authors:** Bin Li, Jianhua Cheng, Hongyun Wang, Sida Zhao, Haibo Zhu, Chuzhong Li, Yazhuo Zhang, Peng Zhao

**Affiliations:** 1 Neurosurgical Department, Beijing Tiantan Hospital, Capital Medical University, No. 119, South Fourth Ring West Road, Fengtai District, Beijing, 100070 China; 2 Department of Cell and Biology, Beijing Neurosurgical Institute, Capital Medical University, No. 119, South Fourth Ring West Road, Fengtai District, Beijing, 100070 China

**Keywords:** Pituitary adenoma, Cavernous sinus invasion, CCNB1 gene, Epithelial–mesenchymal transition, E-Cadherin, N-Cadherin

## Abstract

**Background:**

To investigate the relationship between cyclin B1 (CCNB1) gene expression and cavernous sinus invasion in pituitary adenomas.

**Methods:**

Twenty-four pituitary adenoma tissue samples were examined by RT-qPCR and Western blot to assess the mRNA expression levels and protein levels of CCNB1, E-cadherin and N-cadherin. Correlation analyses between the expression levels of E-cadherin, N-cadherin and CCNB1 were performed. After lentivirus-mediated knockdown of CCNB1 in rat pituitary adenoma cell lines (GH3 and GT1-1), cell function changes were studied. The relationship between CCNB1 and epithelial-mesenchymal transition (EMT) was further verified by animal experiments.

**Results:**

CCNB1 and N-cadherin gene expression were significantly higher in the invasive pituitary adenomas than in the non-invasive pituitary adenomas. Conversely, E-cadherin expression in the invasive pituitary adenomas was significantly lower. CCNB1 gene expression was downregulated in the GH3 and GT1-1 pituitary adenoma cell lines; N-cadherin expression was also decreased, but E-cadherin expression was increased. These results were confirmed in vivo. After downregulation of CCNB1, cell invasion and migration was significantly reduced in Transwell experiments.

**Conclusion:**

High CCNB1 expression in pituitary adenoma affects cavernous sinus invasion through EMT.

## Background

Pituitary adenoma is one of the most common nervous system tumours, accounting for approximately 10–15% of intracranial tumours. The incidence of pituitary adenomas has increased in recent years [1, 2]. Some pituitary adenomas are invasive, leading to the enclosure and compression of important adjacent structures, including the cavernous sinus, making surgery and treatment difficult. Pituitary adenomas that invade the cavernous sinus area account for 6–10% of all pituitary adenomas [3]. These refractory pituitary adenomas adversely affect patient survival and quality of life [4, 5]. The clinical diagnosis and treatment of pituitary adenomas that invade the cavernous sinus are fraught with difficulties and challenges, such as difficulties in preoperative classification, inaccurate predictions of prognosis, incomplete tumour resections, postoperative recurrences and drug resistance. Therefore, exploring the biological characteristics of pituitary adenomas that invade the cavernous sinus and studying the molecular biological mechanism involved in their occurrence and development will help to resolve the abovementioned difficulties in clinical diagnosis and treatment, thus improving the pituitary adenoma cure rate, reducing the recurrence of pituitary adenoma, and improving the prognosis of patients.

Cyclin B1, which is encoded by the CCNB1 gene, belongs to the cyclin family of cell cycle proteins. Previous studies have demonstrated that the CCNB1 gene is highly expressed in pituitary adenomas and is associated with invasiveness, suggesting that the CCNB1 gene plays an important role in the genesis and development of pituitary adenomas [6]. Similarly, CCNB1 was shown to be closely involved in the pathogenesis of pituitary adenomas in Zhang’s research [7].

Our subsequent research found that suppressing the CCNB1 gene can regulate the proliferation and apoptosis of pituitary tumour cells and activate the epithelial-mesenchymal transition (EMT) process. These findings may provide important knowledge for understanding the biological mechanisms of CCNB1 in the development and progression of pituitary adenoma. Furthermore, previous studies have suggested that CCNB1 may be closely related to pituitary adenomas that invade the cavernous sinus. At the same time, the invasion of the cavernous sinus by the pituitary adenoma may be related to the activation of the EMT process.

EMT is a biological process in which epithelial cells lose their epithelial properties and become mesenchymal and invasive. EMT is characterized by alterations in adhesion structure and polarity between epithelial cells, which increases the ability of epithelial cells to deform and migrate [8–10]. EMT is also characterized by the downregulation of epithelial cell markers (E-cadherin) and upregulation of mesenchymal cell markers (N-cadherin) [11]. Many studies have shown that the EMT process has an important impact on the migration, invasion and metastasis of tumour cells [12–14].

The aim of this study was to investigate the relationship between CCNB1 expression levels in and cavernous sinus invasion by pituitary adenomas and to further elucidate the mechanism of cavernous sinus invasion by pituitary adenomas.

## Methods

### Clinical materials and tissue specimens

A total of 24 pituitary adenoma specimens were obtained from September 2017 to December 2018 at Beijing Tiantan Hospital, Capital Medical University. This study was approved by the Beijing Tiantan Hospital Ethics Committee, and written informed consent was obtained from every participant. Patients with pituitary adenoma were divided into the cavernous sinus non-invasion group (N-invasion) and the cavernous sinus invasion group (invasion) according to the Knosp classification by preoperative MRI. Pituitary adenomas classified as Knosp grades 0–2 made up the N-invasion group, and pituitary adenomas classified as Knosp grades 3–4 made up the invasion group. The pituitary adenoma specimens were quickly frozen in liquid nitrogen within 30 min after surgery. All patients were followed up after discharge for 3 months to 1 year.

### Cells and cell culture

The rat pituitary tumour cell line GH3 (American Type Culture Collection, USA) was maintained in F-12K (American Type Culture Collection, USA) supplemented with 15% horse serum (HS) (Gibco, USA) and 2.5% foetal bovine serum (FBS) (Gibco, USA). The mouse pituitary tumour cell line GT1-1 (American Type Culture Collection, USA) was maintained in DMEM/F12 (Gibco, USA) supplemented with 10% foetal bovine serum (FBS) (Gibco, USA). GH3 and GT1-1 cells were cultured in a humidified incubator at 37 °C in 5% CO_2_.

### CCNB1 shRNA recombinant lentiviral vectors

Based on the CCNB1 gene sequence (Gene ID: 25203) in GenBank, primers for CCNB1 shRNA and the negative control were designed and cloned into the pGMLV-SC5 vector. Complementary oligonucleotides were synthesized and paired to form double-stranded DNA after annealing. The double-enzyme linearized lentiviral pGMLV vector-SC5 and the double-stranded DNA fragment were ligated overnight at 16 °C via T4 DNA ligase. The ligated product was transformed into DH5α *E. coli* competent cells, and transformants were picked for colony PCR. The PCR-positive clones were sequenced, and for endotoxin removal, plasmids were extracted using a plasmid extraction kit (QIAGE, Germany).

### Lentivirus packaging and transfection

HEK 293T cells (American Type Culture Collection, USA) were selected for packaging and lentivirus titre measurements. The constructed lentiviral vector and its auxiliary original packaging vector plasmid were co-transfected into 293T cells using HG transgene reagent. After 48 h of cell culture, cell supernatants enriched with lentiviral particles were collected and concentrated to obtain high-titre lentivirus concentrates, and the virus titre was determined and calibrated in 293T cells. The virus-containing solution was diluted by means of the hole dilution method and added to the 293T cells. Two days after transfection, the medium containing the lentivirus was removed and replaced with complete medium. On the fifth day, the number of fluorescent cells in the wells was counted under a fluorescence microscope. Viral titre = number of cells expressing fluorescence gene × dilution factor.

### Infection of target cells with lentiviral particles

GH3 and GT1-1 cells were seeded in 6-well plates at a cell seeding density of 2 × 10^5^ cells/well. When the cell density reached 40–50%, each group was treated with lentiviruses and appropriate concentrations of polybrene. The experimental groups were the non-infected (full control, FC) group, control shRNA-infected (negative control, NC) group, and CCNB1 shRNA-infected (shRNA) group. Next, 2 × 10^6^ transducing units (TU) per well of the recombinant lentivirus were added to the plate and incubated at 37 °C in 5% CO_2_ for 3 days. The transfection efficiency was determined daily using a fluorescence microscope.

### RNA extraction and RT-qPCR

Total RNA was extracted from human pituitary adenoma tissues and from GH3 and GT1-1 cells using the RNeasy Mini Kit (QIAGEN, Germany) according to the manufacturer’s instructions. Reverse transcription was performed with the High-Capacity cDNA Reverse Transcription Kit (Thermo Fisher Scientific, USA) according to the manufacturer’s instructions. Primers were designed and synthesized by GenBank to locate gene sequences (Table 1). Quantitative PCR (qPCR) was performed using PowerUp™ SYBR™ Green Master Mix (Thermo Fisher Scientific, USA) and Pharmaceutical Analytics QuantStudio™ 5 Real-Time PCR System (Thermo Fisher Scientific, USA). The relative quantities of each gene were analysed by 2^−∆∆Ct^.

**Table 1:** Sequences of the primers used for RT-qPCR

Gene	Forward primer	Reverse primer
CCNB1 (human)	TGTTGGTTTCTGCTGGGTGT	TGCCATGTTGATCTTCGCCT
E-Cadherin (human)	GCTGGACCGAGAGAGTTTCC	CAAAATCCAAGCCCGTGGTG
N-Cadherin (human)	GGGAAATGGAAACTTGATGGCA	CAGTTGCTAAACTTCACTGAAAGGA
GAPDH (human)	AATGGGCAGCCGTTAGGAAA	GCCCAATACGACCAAATCAGAG
CCNB1 (rat)	ATCGGTTCATGCAGGACAGT	TGGAGGGTACATCTCCTCGT
E-Cadherin (rat)	TTGAGAATGAGGTCGGTGCC	ATCCAAGCCCTTGGCTGTTT
N-Cadherin (rat)	CACCCGGCTTAAGGGTGATT	CGATCCTGTCTACGTCGGTG
GAPDH (rat)	AGTGCCAGCCTCGTCTCATA	GACTGTGCCGTTGAACTTGC

### Protein extraction and western blot

Tumour tissues and cells were lysed with RIPA buffer (Applygen, China) containing a protease inhibitor (Applygen, China) cocktail. The protein concentration was determined using a BCA Protein Assay kit (Thermo Fisher Scientific, USA). Proteins were separated on 10% SDS-PAGE and transferred to PVDF membranes (Millipore, USA). The membranes were treated with 5% skimmed milk in TBST for 1 h at room temperature and then incubated with primary antibodies (Table 2) at 4 °C overnight. After being washed with TBST three times (5 min each), the membrane was incubated with secondary antibodies (Table 2) for 1 h at room temperature and then washed with TBST three times (5 min each). The Western Blotting Luminol Reagent (Santa Cruz Biotechnology, USA) was used for detection. GAPDH was used as the internal control, and the grey values of the protein bands were quantified with Image Pro Plus software.

**Table 2:** The antibodies used in the experiments

Antibody	No.	Company	Country
Anti-Cyclin B1 antibody	ab2949	Abcam	Britain
Anti-E-Cadherin antibody	K003568P	Solarbio	China
Anti-N-Cadherin antibody	ab18203	Abcam	Britain
Anti-GAPDH antibody	K200057M	Solarbio	China
Anti-Rabbit antibody	ZB-2301	ZSGB-BIO	China
Anti-Mouse antibody	ZB-2305	ZSGB-BIO	China

### Transwell migration and invasion assay

Cell migration and invasion were determined using Transwell chambers containing membranes with 8-μm pores in 24-well culture plates (Corning, USA). The experimental groups were the non-infected (FC) group, control shRNA-infected (NC), and CCNB1 shRNA-infected (shRNA) group. According to the manufacturer’s instructions, the Corning Matrigel basement membrane matrix (Corning, USA) was thawed on ice at 4 °C overnight. The Matrigel matrix was diluted in serum-free medium to a final concentration of 200 μg/mL. Next, 100 μL of the diluted Matrigel matrix was carefully added to the centre of each Transwell insert for invasion assays. The plate was incubated at 37 °C for 1 h to allow the Matrigel matrix to form a gel. Transwell inserts that were intended for migration experiments were not coated with Matrigel matrix. A total of 150 μL of the cell suspension was added into the upper chamber of each Transwell. The final cell density was 7.5 × 10^4^ cells/well in serum-free medium. A total of 800 μL of culture medium containing 10% FBS as the chemoattractant was added to the lower chamber of each well. The cells were cultured in a humidified incubator at 37 °C with 5% CO_2_ overnight. The cells on the lower surface of the membrane were then stained with crystal violet for 10 min. The Transwell inserts were washed twice with PBS to remove unbound crystal violet and then air-dried. The invaded and migrated cells were observed and imaged under a microscope (200×). Quantification was achieved by averaging the number of cells counted in five randomized fields.

### Xenograft experiments and immunohistochemistry

For in vivo assays, a GH3 cell xenograft model was performed. This study was approved by the Beijing Tiantan Hospital Ethics Committee. Twenty male BALB/c nude mice (6 weeks old) were randomly divided into two groups, and the right axilla of each mouse was subcutaneously injected with 3 × 10^6^ GH3 cells transfected with either CCNB1 shRNA or scramble-shRNA in serum-free medium. Four weeks after the cells were injected, the mice were euthanized, and the tumour volumes and weights were measured. H&E staining of all specimens was performed to evaluate tumour content and quality. Immunohistochemical analyses with primary antibodies (Table 2) were performed on the sections from all TMAs using the Leica BOND-III automated, random, and continuous-access slide staining system (Leica Biosystems, Germany) according to the manufacturer’s instructions. The percentage of positively stained cells was quantified using Image Pro Plus software according to the IOD to Area ratio at 400× magnification.

### Statistical analysis

We used SPSS 25.0 statistical software for statistical evaluations. The results are expressed as the mean ± SEM. Statistical analysis of the data was performed using one-way ANOVA or independent two-sample t tests (based on the grouping situation). Correlations were evaluated by means of Pearson’s correlative analysis. p < 0.05 was considered statistically significant.

## Results

### Patients and tumour characteristics

**Fig. 1:**
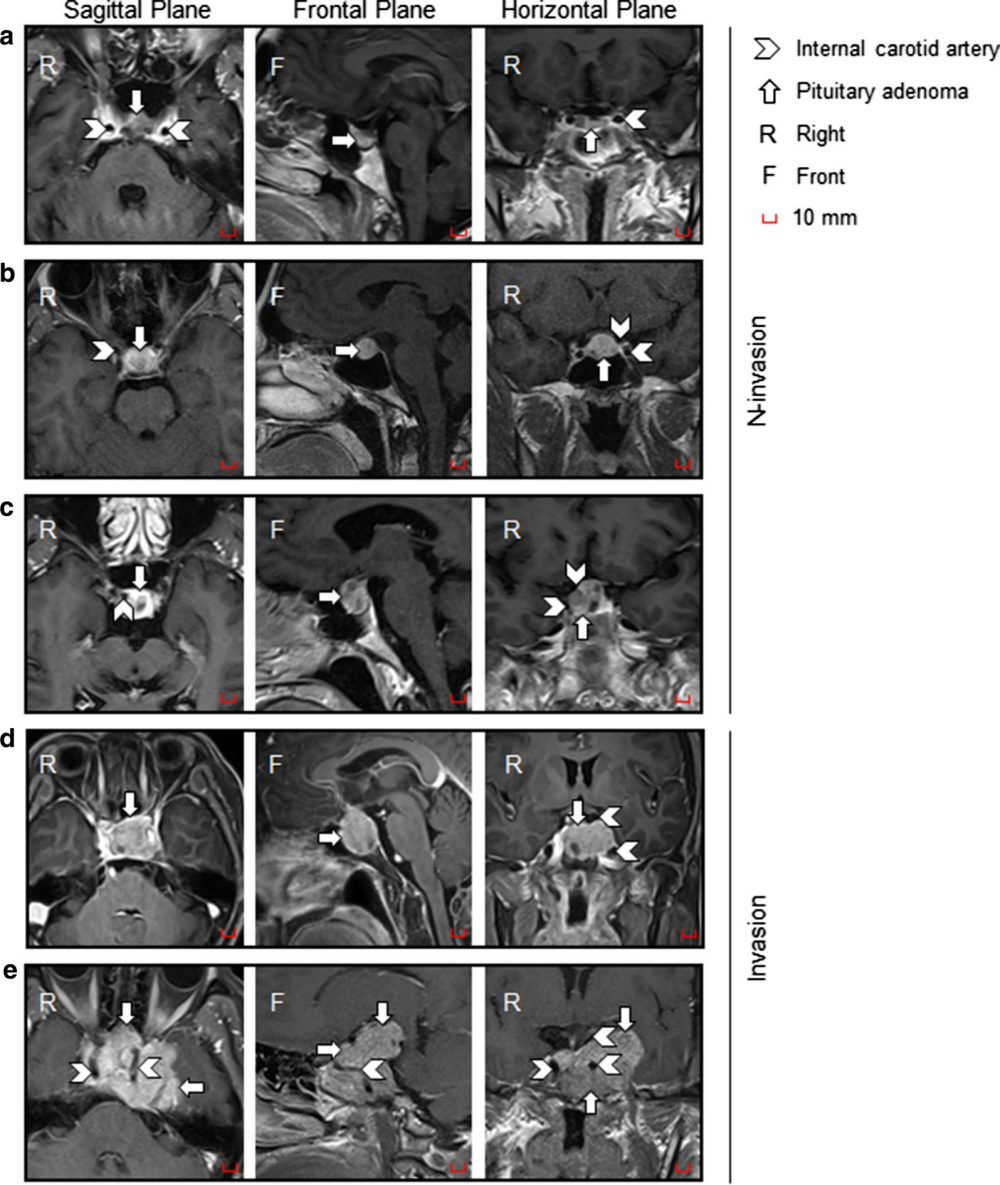
The MR performance of non-invasive and invasive pituitary adenomas. **a** Knosp grade 0. **b** Knosp grade 1. **c** Knosp grade 2. **d** Knosp grade 3. **e** Knosp grade 4. Pituitary adenomas classified as Knosp grades 0–2 constitute the N-invasion group, and pituitary adenomas classified as Knosp grades 3–4 constitute the invasion group (*N-invasion* non-invasive pituitary adenomas, *Invasion* invasive pituitary adenomas)

Twenty-four patients (8 women and 16 men) with pituitary adenoma were included in our study. The mean age was 48.6 years (range 31–69 years) at the time of surgery. The patients with pituitary adenoma were divided into the cavernous sinus non-invasion group (N-invasion) and the cavernous sinus invasion group (invasion) according to the Knosp classification by preoperative MRI. Pituitary adenomas classified as Knosp grades 0–2 made up the N-invasion group, and pituitary adenomas classified as Knosp grades 3–4 made up the invasion group (Fig. 1a–e). Based on preoperative endocrinological tests and postoperative pathological results, there were 4 functional adenomas (two growth hormone (GH)-producing adenomas, one adrenocorticotropic hormone (ACTH)-producing adenoma, and one prolactinoma that was refractory to conservative treatment), and 20 non-functional adenomas. Cavernous sinus invasion was not found to correlate with patient age, sex, or hormone secretion type.

### CCNB1 and N-cadherin mRNA and protein expression levels were higher, and E-cadherin expression was lower in the invasive pituitary adenomas

**Fig. 2:**
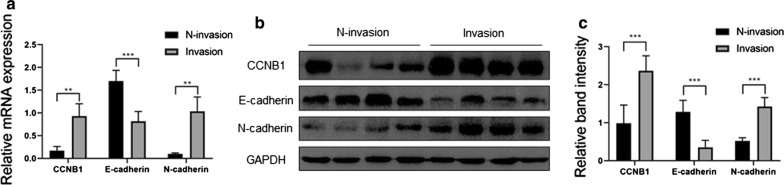
The expression of CCNB1, E-cadherin and N-cadherin in pituitary adenomas at the RNA and protein levels. **a** Quantification of mRNA expression levels of CCNB1, E-cadherin and N-cadherin by RT-qPCR in invasive and non-invasive pituitary adenomas. **b**, **c** Quantification of the protein expression levels of CCNB1, E-cadherin and N-cadherin by Western blot in invasive and non-invasive pituitary adenomas. Blots were subjected to the same exposure conditions. Band intensities were quantified and standardized to GAPDH. **p < 0.01; ***p < 0.001 (*N-invasion* non-invasive pituitary adenomas, *Invasion* invasive pituitary adenomas)

The RT-qPCR results showed that CCNB1 (p < 0.01) and N-cadherin (p < 0.01) were overexpressed in the invasive pituitary adenomas compared to the non-invasive pituitary adenomas and that E-cadherin (p < 0.001) expression was significantly decreased (Fig. 2a). The Western blot (WB) results also showed that CCNB1 (p < 0.001) and N-cadherin (p < 0.001) were overexpressed in the invasive pituitary adenomas compared to the non-invasive pituitary adenomas and that E-cadherin (p < 0.001) expression was significantly decreased (Fig. 2b, c).

### Negative correlation between CCNB1 and E-cadherin expression and positive correlation between CCNB1 and N-cadherin expression in pituitary adenomas

**Fig. 3:**
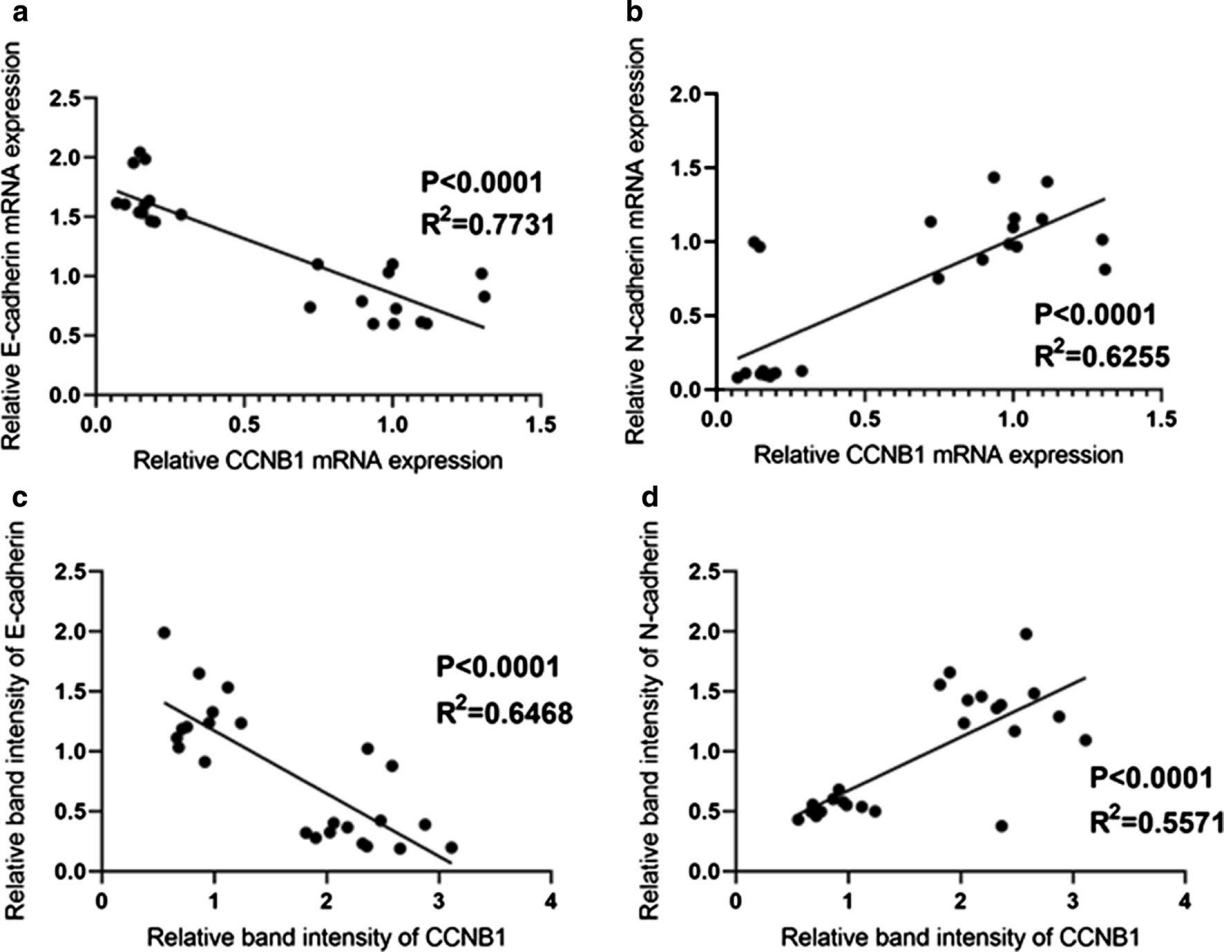
The correlation between CCNB1 and E-cadherin expression and between CCNB1 and N-cadherin expression in human pituitary adenomas. The mRNA expression of CCNB1 was negatively correlated with that of E-cadherin (**a**) but positively correlated with that of N-cadherin (**b**) in human pituitary adenomas. The protein expression of CCNB1 was negatively correlated with that of E-cadherin (**c**) but positively correlated with that of N-cadherin (**d**) in human pituitary adenomas. p < 0.0001 for all

The correlations between CCNB1 and E-cadherin expression and between CCNB1 and N-cadherin expression in pituitary adenomas were analysed. At the mRNA level, a negative correlation between CCNB1 and E-cadherin (p < 0.0001, R^2^ = 0.7731) (Fig. 3a) and a positive correlation between CCNB1 and N-cadherin (p < 0.0001, R^2^ = 0.6255) were observed (Fig. 3b). At the protein level, a negative correlation between CCNB1 and E-cadherin (p < 0.0001, R2 = 0.6468) (Fig. 3c) and a positive correlation between CCNB1 and N-cadherin (p < 0.0001, R2 = 0.5571) were observed (Fig. 3d).

### CCNB1 expression by lentiviral transduction downregulates CCNB1 expression

**Fig. 4:**
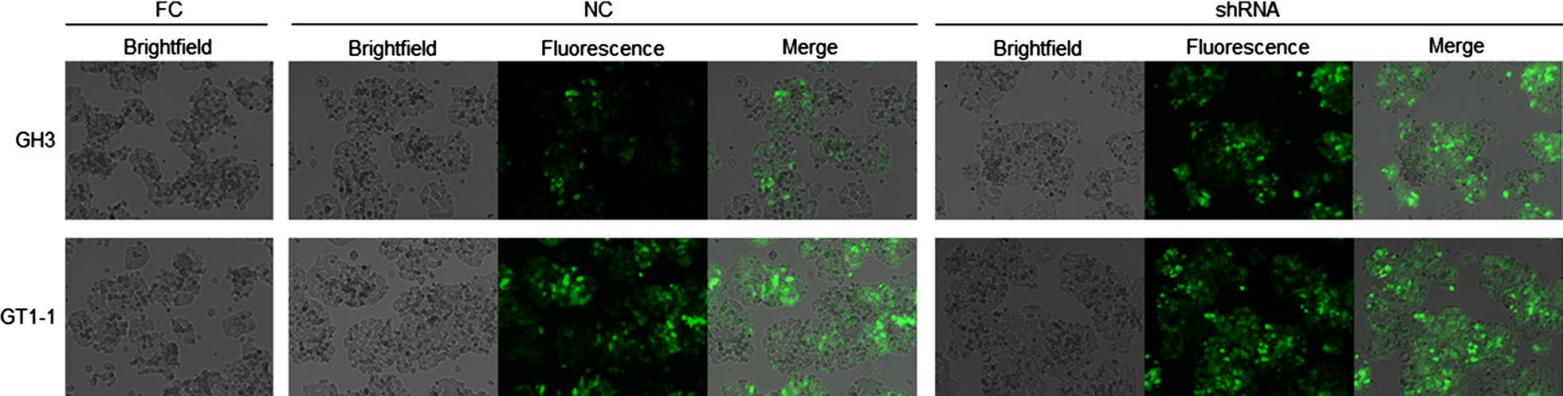
Brightfield, fluorescence and merged images of the FC, NC, and shRNA groups of the GH3 and GT1-1 cell lines after lentivirus transfection (*FC* full control group (no treatment), *NC* negative control scramble-shRNA group, *shRNA* CCNB1-shRNA group)

**Fig. 5:**
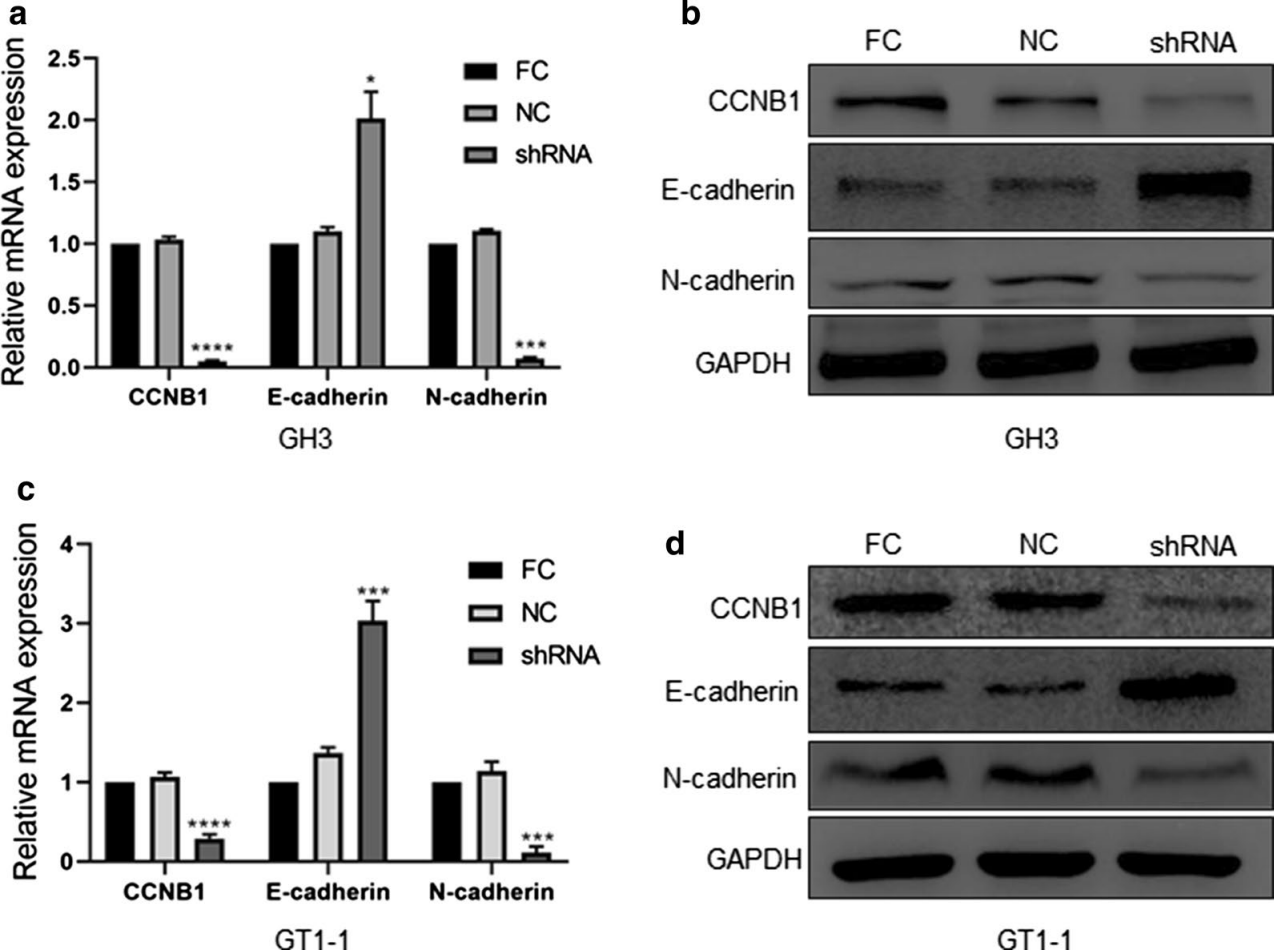
The expression of CCNB1, E-cadherin and N-cadherin after lentivirus transfection. **a**, **c** Quantification of the mRNA expression levels of CCNB1, E-cadherin and N-cadherin in the GH3 and GT1-1 cell lines after lentivirus transfection. **b**, **d** The protein expression levels of CCNB1, E-cadherin, N-cadherin and GAPDH in the GH3 and GT1-1 cell lines after lentivirus transfection. *p < 0.05; ***p < 0.001; ****p < 0.0001 (*FC* full control group (no treatment), *NC* negative control scramble-shRNA group, *shRNA* CCNB1-shRNA group)

CCNB1 shRNA was successfully packaged by lentivirus. We examined the infection efficiency by means of fluorescence images of GFP-positive GH3 cells after lentivirus infection (Fig. 4). The lentivirus transfection efficiency reached 70% by cell counting under a fluorescence microscope. The silencing effect was validated via RT-qPCR and WB. The results showed that both the mRNA and protein levels of CCNB1 were significantly reduced (p < 0.0001) in the GH3 and GT1-1 cell lines (Fig. 5a–d).

### Downregulation of CCNB1 inhibits the EMT process in pituitary adenoma cells

CCNB1 inhibition of EMT processes was confirmed at the RNA and protein levels. The RT-qPCR results showed that the expression of the epithelial cell marker E-cadherin was increased significantly in GH3 cells (p < 0.05) and GT1-1 cells (p < 0.001) after CCNB1 was knocked down. Conversely, the levels of mesothelial cell marker N-cadherin were significantly decreased in GH3 cells (p < 0.001) and GT1-1 cells (p < 0.001) (Fig. 5a, c). The WB results showed that E-cadherin was increased significantly in GH3 and GT1-1 cells after CCNB1 was knocked down. Conversely, the levels of N-cadherin were significantly decreased in GH3 and GT1-1 cells (Fig. 5b, d). These results suggest that CCNB1 can induce EMT in pituitary adenoma cells.

### Downregulation of CCNB1 inhibits pituitary cell migration and invasion

**Fig. 6:**
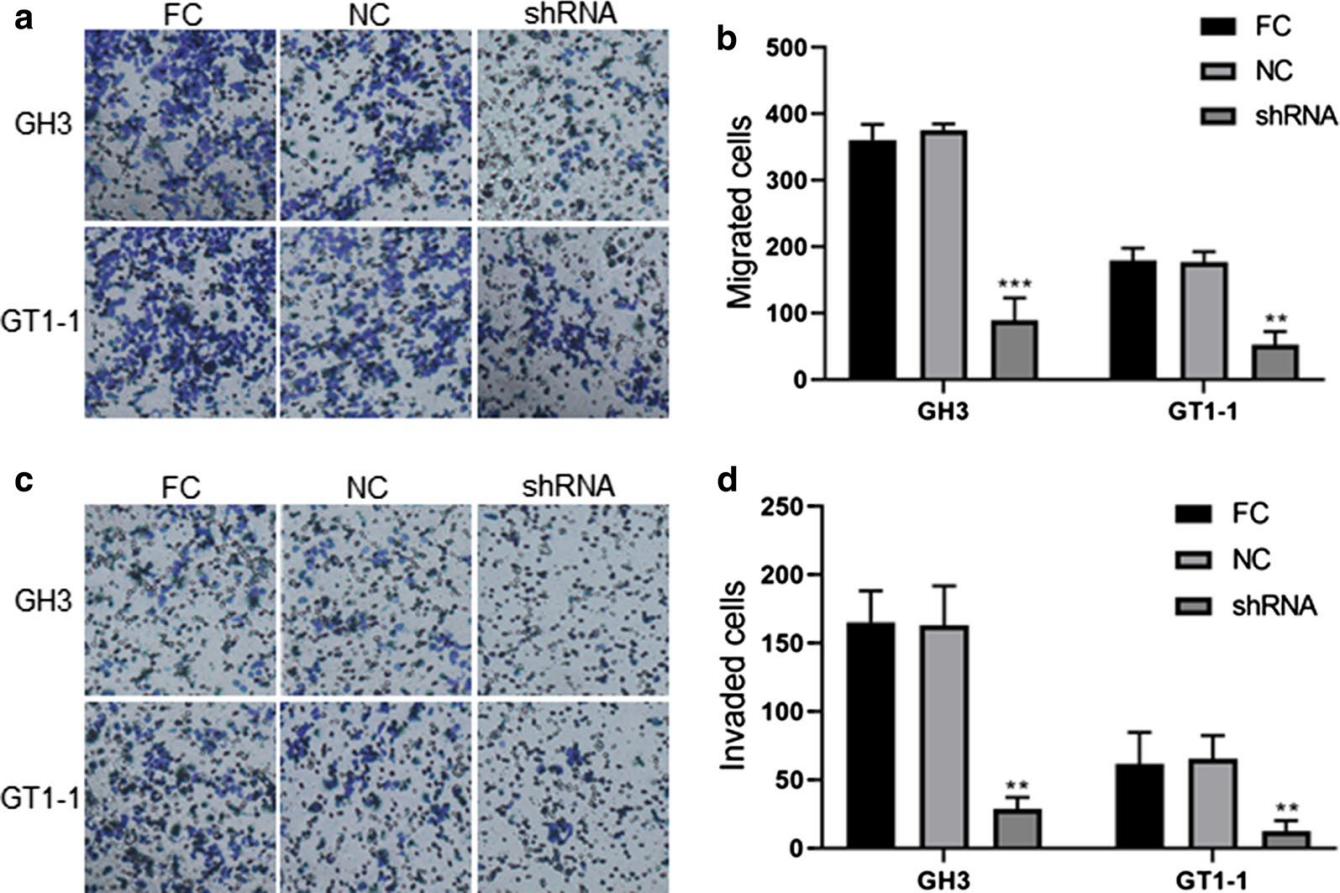
Downregulation inhibits pituitary adenoma cell migration and invasion of (×200). **a**, **b** Transwell migration assays were performed to assess the migrative potential of the GH3 and GT1-1 cell lines after lentivirus transfection. **c**, **d** Transwell invasion assays were performed to assess the invasive potential in the GH3 and GT1-1 cell lines after lentivirus transfection. **p < 0.01; ***p < 0.001 (*FC* full control group (no treatment), *NC* negative control scramble-shRNA group, *shRNA* CCNB1-shRNA group)

We examined the effects of CCNB1 knockdown on cell migration and invasion using Transwell assays. The results showed that after CCNB1 was knocked down, cell migration decreased in GH3 (p < 0.001) and GT1-1 (p < 0.01) cells (Fig. 6a, b). Additionally, the invasive ability of GH3 (p < 0.01) and GT1-1 (p < 0.01) cells also decreased (Fig. 6c, d). These results indicated that the downregulation of CCNB1 inhibited the migration and invasion of pituitary adenoma cells.

### Downregulation of CCNB1 impairs tumourigenesis and inhibits the EMT process in pituitary adenoma cells in vivo

**Fig. 7:**
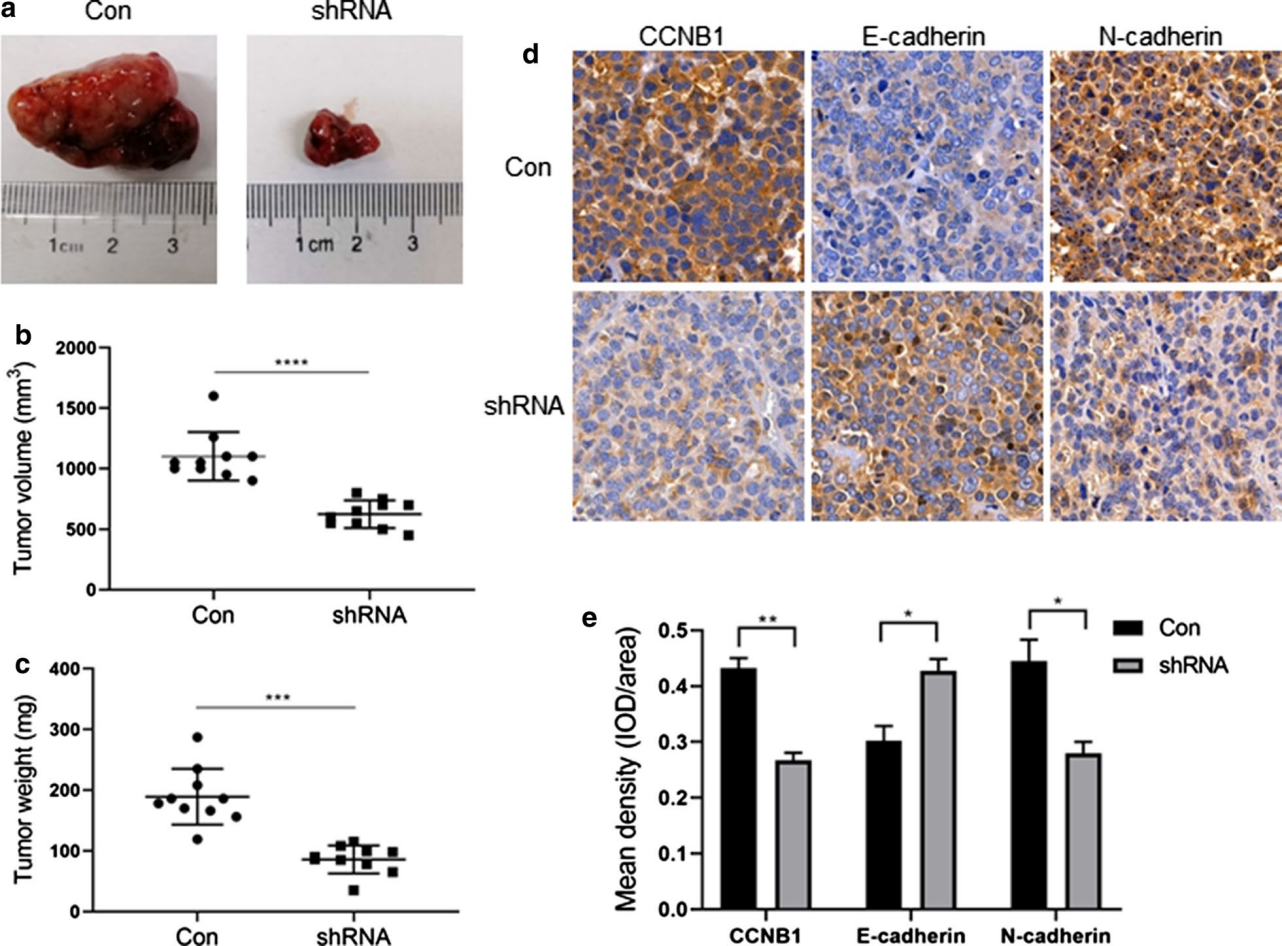
Tumourigenesis and CCNB1, E-cadherin and N-cadherin expression in vivo. **a** Representative images of tumours in the nude mouse GH3 xenograft model. **b** Statistical analysis of xenograft tumour volume. **c** Statistical analysis of xenograft tumour weight. **d** Representative immunohistochemistry images of xenograft tumour tissues with CCNB1, E-cadherin and N-cadherin staining (×400). **e** The percentage of cells that stained positive was quantified using Image Pro Plus software according to the IOD to Area ratio at ×400 magnification. *p < 0.05; **p < 0.01; ***p < 0.001; ****p < 0.0001 (*Con* scramble-shRNA-transfected GH3 cell group, *shRNA* CCNB1 shRNA-transfected GH3 cell group)

Next, we examined whether CCNB1 regulates tumourigenesis and the EMT process in pituitary adenoma cells in vivo. The results showed that CCNB1 silencing significantly reduced the xenograft tumour volume (p < 0.0001) and weight (p < 0.001) in vivo (Fig. 7a–c). Immunohistochemical analysis showed that after CCNB1 was silenced (p < 0.01), the expression of E-cadherin increased (p < 0.05), and the expression of N-cadherin decreased (p < 0.05) (Fig. 7d, e). These results suggested that the downregulation of CCNB1 impaired tumourigenesis and inhibited the EMT process in pituitary adenoma cells in vivo.

## Discussion

Cavernous sinus invasion is an important factor that affects the prognosis of patients with pituitary adenomas [15, 16]. Invasive pituitary adenoma is challenging and is a hot topic in the field of neurological tumours. In the most recent study, a significant positive association between tumour invasion and ESM-1 expression in null cell adenoma was reported [17]. Drummond found that silent tumours seem to be more aggressive than their secreting counterparts and have a higher recurrence rate [18]. However, the biological mechanism underlying the invasiveness of pituitary adenomas is poorly understood.

The CCNB1 gene is overexpressed in many human tumours and is involved in the proliferation, invasion, metastasis and recurrence of various tumours [19–21]. In addition, CCNB1 can also serve as a prognostic biomarker for oestrogen receptor positive (ER+) breast cancer [22]. Some studies have shown that CCNB1 can affect the development of pituitary tumours through the cell cycle [23, 24]. A previous study by our team found that the CCNB1 gene is highly expressed in pituitary adenomas and correlates with invasiveness [25]. The specific mechanism by which CCNB1 affects the biological behaviour of pituitary adenoma invasion is unclear. In particular, the clear mechanism by which pituitary adenomas invade the cavernous sinus has not been reported in the literature. In our study, CCNB1 gene expression was significantly higher in invasive pituitary adenomas than in non-invasive pituitary adenomas. This finding suggests that the CCNB1 gene has effects on the invasiveness of pituitary tumours.

The EMT process was originally described in the context of embryonic development [26]. During embryonic development, the EMT process is involved in the development of the pituitary gland [27]. Pituitary stem/progenitor cells migrate by means of the EMT process, and the pituitary gland develops. In adults, EMT is involved in multiple processes, such as wound healing, tissue fibrosis and cancer metastasis [28]. Studies have shown that the upregulation of EMT can lead to the invasion, metastasis and poor prognosis of breast cancer [29]. In addition, studies have confirmed that the regulation of EMT can affect the invasion and metastasis of colorectal cancer [30]. In thyroid carcinomas, EMT markers, including N-cadherin, Vimentin and Snail, are regulated by IL13RA2 [31].

There are many markers that are associated with EMT. David reported that the markers Vimentin, Smooth-Muscle-Actin, N-cadherin, Cadherin-11, E-cadherin, Cytokeratins and β-catenin are related to EMT [32]. According to Peng’s study, the markers associated with EMT are E-cadherin, N-cadherin, Vimentin, Snail, Slug, Twist, Fibronectin, Occludin and Claudin [33]. By analysing the expression of E-cadherin and N-cadherin, Lei confirmed that LncRNA TUG1 affects EMT in papillary thyroid carcinoma [34]. Wang also examined E-cadherin and N-cadherin levels and confirmed that HPIP promotes EMT in thyroid cancer cells [35]. In our study, the expression of E-cadherin and N-cadherin was also examined. Our analysis of multiple reports showed that E-cadherin and N-cadherin are often used in EMT-related research. These two indicators are negatively correlated and can indicate EMT-related issues.

In pituitary adenomas, EMT plays a very important role in the occurrence and development of pituitary tumours [36]. Changes in EMT markers constitute an important indicator of tumour progression, bone destruction and endocrine functions [37]. A decrease in the tumour cell E-cadherin level can turn a non-invasive tumour into a highly aggressive one. The presence of EMT markers in metastatic sites and in transformed tumour cells has been confirmed. Our study showed that the expression of N-cadherin was significantly higher in invasive pituitary adenomas than in non-invasive pituitary adenomas. Conversely, the expression of E-cadherin in invasive pituitary adenomas was significantly lower. A negative correlation between CCNB1 and E-cadherin and a positive correlation between CCNB1 and N-cadherin were observed. These results indicate that the invasion and migration mechanism of pituitary adenoma may be related to the EMT process. To further clarify this mechanism, the shRNA method was used to silence the CCNB1 gene in pituitary tumour cells. The results showed that the expression of important EMT markers changed with the downregulation of CCNB1 gene expression, suggesting that the CCNB1 gene may induce cell migration and invasion by promoting the EMT process and increasing cell transformation. Transwell analysis confirms this view. Finally, our study revealed that the downregulation of CCNB1 impaired tumourigenesis and inhibited the EMT process in pituitary adenoma cells in vivo. These results strongly support the notion that CCNB1 affects the migration and invasion of pituitary adenomas by its involvement in the EMT process.

In terms of treatment, Singh has summarized clinical interventions that are focused on targeting various aspects of EMT and their contributions to preventing cancer dissemination [38]. Using in vivo and in vitro studies, we demonstrated that CCNB1 promotes the migration and invasion of pituitary tumour cells be its involvement in EMT. The correlation between the CCNB1 gene and EMT may be a potential target for the diagnosis and treatment of pituitary adenomas.

The limitation of this study is the number of pituitary tumour samples, which is small. In the next study, we will expand the number of samples for further verification.

## Conclusion

The high expression of CCNB1 in pituitary adenoma affects cavernous sinus invasion by its involvement in EMT. The correlation between the CCNB1 gene and EMT may be a potential target for the diagnosis and treatment of pituitary adenomas. Further clarification of the mechanism of CCNB1 actions in EMT may provide a new strategy for the treatment of pituitary adenomas.

## Data Availability

The authors can confirm that all relevant data and materials are available upon request from the authors.

## Abbreviations

CCNB1: cyclin B1
EMT: epithelial–mesenchymal transition
GH: growth hormone
ACTH: adrenocorticotropic hormone
